# Transcriptome reprogramming due to the introduction of a barley telosome into bread wheat affects more barley genes than wheat

**DOI:** 10.1111/pbi.12913

**Published:** 2018-04-13

**Authors:** Elodie Rey, Michael Abrouk, Gabriel Keeble‐Gagnère, Miroslava Karafiátová, Jan Vrána, Sandrine Balzergue, Ludivine Soubigou‐Taconnat, Véronique Brunaud, Marie‐Laure Martin‐Magniette, Takashi R. Endo, Jan Bartoš, International Wheat Genome Sequencing Consortium (IWGSC), Rudi Appels, Jaroslav Doležel

**Affiliations:** ^1^ Institute of Experimental Botany Centre of the Region Haná for Biotechnological and Agricultural Research Olomouc Czech Republic; ^2^ Agriculture Research Victoria Department of Economic Development Jobs Transport and Resources AgriBio Bundoora VIC 3083 Australia; ^3^ Institute of Plant Sciences Paris Saclay IPS2 CNRS INRA Université Paris‐Sud Université Evry Université Paris‐Saclay Orsay France; ^4^ Institute of Plant Sciences Paris‐Saclay IPS2 Paris Diderot Sorbonne Paris‐Cité Orsay France; ^5^ IRHS Université d'Angers INRA AGROCAMPUS‐Ouest SFR4207 QUASAV Université Bretagne Loire Beaucouzé France; ^6^ UMR MIA‐Paris AgroParisTech INRA Université Paris‐Saclay Paris France; ^7^ Department of Plant Life Science Faculty of Agriculture Ryukoku University Shiga Japan; ^8^ Murdoch University Perth WA Australia

**Keywords:** alien introgression, RNA‐seq, gene transcription, transcriptome modification, chromosomal rearrangement, deletion

## Abstract

Despite a long history, the production of useful alien introgression lines in wheat remains difficult mainly due to linkage drag and incomplete genetic compensation. In addition, little is known about the molecular mechanisms underlying the impact of foreign chromatin on plant phenotype. Here, a comparison of the transcriptomes of barley, wheat and a wheat–barley 7HL addition line allowed the transcriptional impact both on 7HL genes of a non‐native genetic background and on the wheat gene complement as a result of the presence of 7HL to be assessed. Some 42% (389/923) of the 7HL genes assayed were differentially transcribed, which was the case for only 3% (960/35 301) of the wheat gene complement. The absence of any transcript in the addition line of a suite of chromosome 7A genes implied the presence of a 36 Mbp deletion at the distal end of the 7AL arm; this deletion was found to be in common across the full set of Chinese Spring/Betzes barley addition lines. The remaining differentially transcribed wheat genes were distributed across the whole genome. The up‐regulated barley genes were mostly located in the proximal part of the 7HL arm, while the down‐regulated ones were concentrated in the distal part; as a result, genes encoding basal cellular functions tended to be transcribed, while those encoding specific functions were suppressed. An insight has been gained into gene transcription in an alien introgression line, thereby providing a basis for understanding the interactions between wheat and exotic genes in introgression materials.

## Introduction

Bread wheat (*Triticum aestivum*) and barley (*Hordeum vulgare*) are the two leading temperate small‐grained cereals, and together supply, directly or indirectly, a major proportion of our calorific requirements. The major breeding priority over many decades has been to increase their productivity, and this goal was growing in importance in the face of a continuing expansion in the size of the human population, changes in the dietary habits and the threat of impending climate change (Curtis and Halford, 2014). A key means to meet this challenge is to extend the genetic diversity available for breeding, and one of the best ways to achieve this is likely to be to unlock the largely untapped diversity harboured by the crops’ wild and cultivated relatives (Brozynska *et al*., 2016; Feuillet *et al*., 2008; Mondal *et al*., 2016). In wheat, continuing technological advances have, over many years, improved the success rate of wide hybridization (Blakeslee, 1937; Kruse, 1973; Mujeeb‐Kazi, 1995; Murashige and Skoog, 1962), while progress in inducing introgression has succeeded in introducing subchromosomal segments of exotic origin (Endo, 1988, 2007; Griffiths *et al*., 2006; Jiang *et al*., 1993; Riley and Chapman, 1958). Many of these can be readily visualized at both the cytogenetic and the genetic levels (Fedak, 1999; Gupta *et al*., 1999; Rayburn and Gill, 1985; Rey *et al*., 2015; Yamamoto and Mukai, 1989). A few introgression products have made positive contributions to grain yield, resistance to disease and tolerance of abiotic stress (Börner *et al*., 2015; Fatih, 1983; Friebe *et al*., 1996; Mondal *et al*., 2016; Reynolds *et al*., 2001; Wulff and Moscou, 2014), but the majority have had little impact on wheat improvement, as their presence has been associated with either a yield penalty and/or an end product defect.

While the long‐standing assumption has been that the poor performance of introgression lines is due to linkage drag, together with—in the case where an exotic chromatin segment replaces its wheat homeolog—a less than perfect compensation for the loss of wheat genes, there is some evidence that gene interactions between the recipient and the donor genes are also implicated. For example, the suppression of a gene determining resistance to a pathogen has been shown to be caused by its interaction with the recipient genome (Chen *et al*., 2005; Kerber and Aung, 1999; Kerber and Dyck, 1973), in some cases shown to be the result of post‐translational modifications being imposed on the donor gene product (Stirnweis *et al*., 2014). According to Hurni *et al*. (2014), deleterious effects can also result from the formation of a heteromeric complex involving donor and recipient gene products.

The set of wheat–barley disomic (Islam *et al*., 1981) and ditelosomic (Islam, 1983) addition lines was bred from a cross between the wheat cultivar Chinese Spring (CS) and the barley cultivar Betzes (B). A transcriptomic analysis of the six available CS/B disomic chromosome addition lines (the one involving 1H is nonviable) (Cho *et al*., 2006) has succeeded in allocating the chromosomal origin of 1787 barley transcripts, but this represents only a limited proportion of the full complement of transcripts, estimated to number than 39 000 (Mascher *et al*., 2017). Here, a line in which the barley 7HL telosome is maintained in a wheat genetic background has been used as a model to characterize interactions between the gene content of the host wheat genome and that of the barley telosome, based on the use of a high capacity transcriptome sequencing platform.

## Results

### Gene transcription analysis

The RNA‐seq procedure yielded on average 14.6 million paired‐end mapped reads per sample, of which 98.78 ± 0.81% proved to be assignable to a genomic site (Table S1). Only a small proportion of the reads (2.41% in B, <1% in CS and CS + 7HL) mapped to an unexpected location. The RSEM analysis revealed that 35 301 wheat genes were transcribed by either CS or CS + 7HL at >1 count per million mapped reads, while the equivalent number of 7HL genes located in the genomic window 339–656 Mbp was 923. Of the 35 301 wheat genes, the vast majority (34 341, 97.3%) were not transcribed differentially between CS and CS + 7HL, still leaving 960 which were differentially transcribed. Of these latter genes, 509 were down‐regulated (transcript abundance lower in CS + 7HL than in CS) and 451 were up‐regulated. The parallel comparison of B *vs* CS + 7HL revealed that of the 923 7HL genes, 389 (42.1%) were differentially transcribed, comprising 233 down‐regulated (transcript abundance lower in CS + 7HL than in B) and 156 up‐regulated (Table 1). These greatly differing proportions of differential transcription (DT) implied that the effect of a non‐native genetic background was much greater than the effect of the presence of an exotic telosome.

**Table 1:** The differential transcription (DT) in CS + 7HL of both wheat and barley (7HL) genes

	Genes	
	Barley 7HL	Wheat
Transcribed (>1 Counts per million (CPM))	923	35 301
Not‐DT	534 (57.85%)	34 341 (97.28%)
DT	389 (42.14%)	960 (2.72%)
Up‐regulated	156 (16.90%)	451 (1.28%)
Down‐regulated	233 (25.24%)	509 (1.44%)

### Distribution and impact of DT

**Figure 1:**
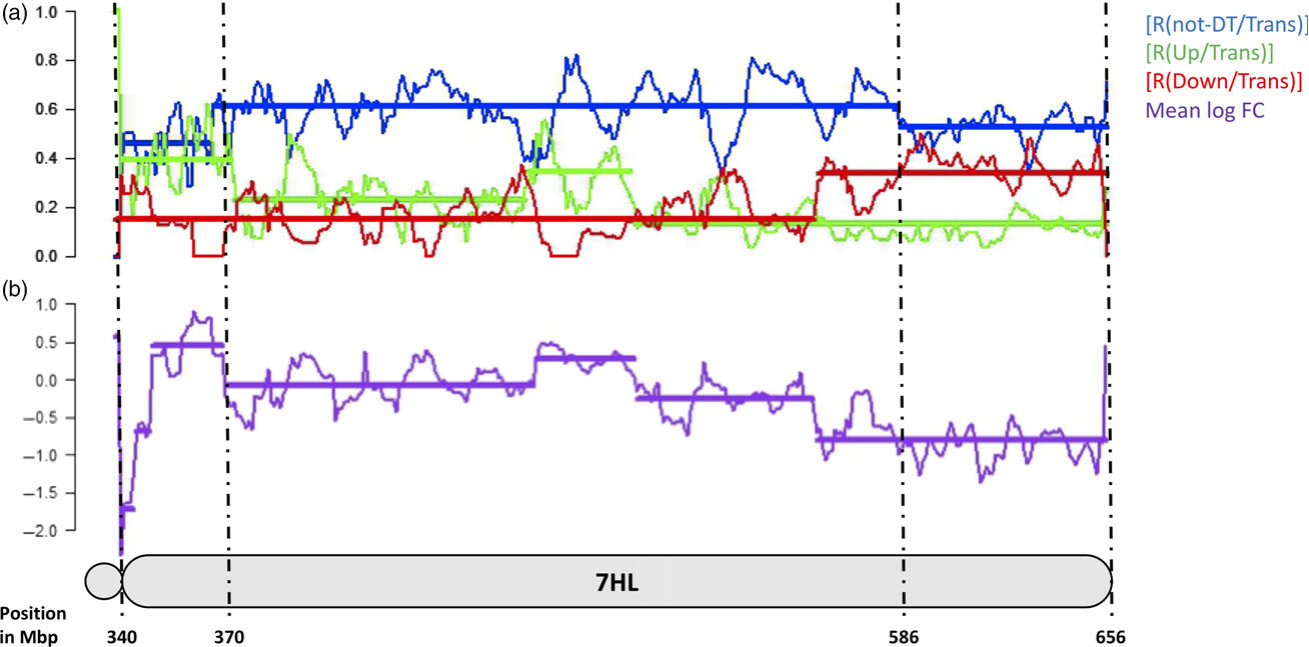
The chromosomal spread of 7HL genes showing altered transcription in CS + 7HL. (a) The ratio of not‐DT to transcribed genes [R(not‐DT/Trans)], the ratio of up‐regulated to transcribed genes [R(Up/Trans)], the ratio of down‐regulated to transcribed genes [R(Down/Trans)]. (b) The mean log FC in CS + 7HL and B along the 7HL chromosome. Each segment is represented by a horizontal bar along each measure (change point positions given in Mbp as positions on the 7H pseudomolecule.

**Figure 2:**
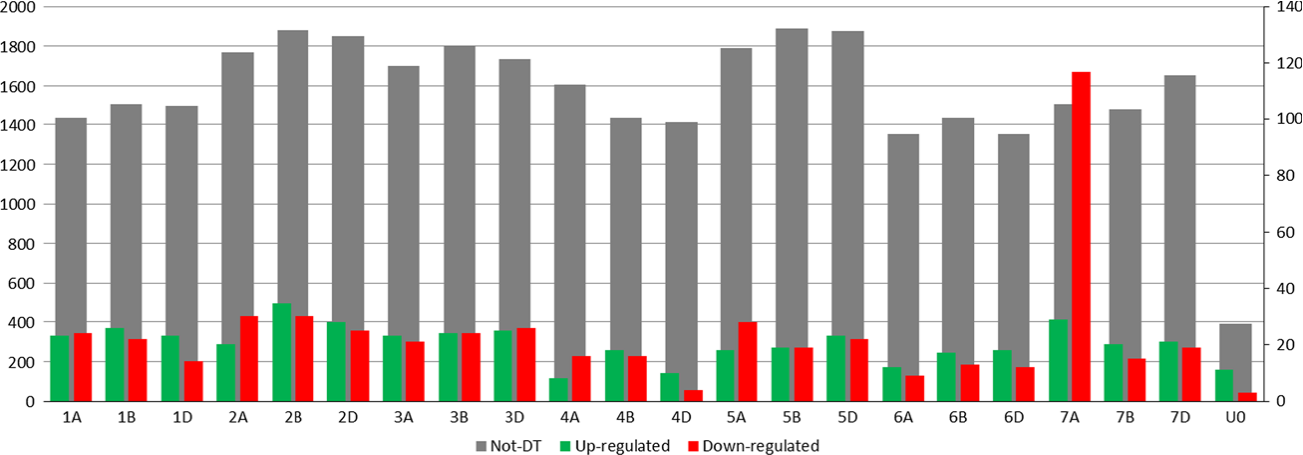
The distribution of not‐DT, down‐ and up‐regulated genes across the 21 chromosomes of wheat in CS + 7HL. The number of not‐DT genes (grey) is given on the left scale, while down‐ (red) and up‐regulated genes (green) ones are represented on the right scale. Not‐DT, up‐ and down‐regulated genes belonging to unanchored scaffolds in the genome assembly have also been represented under the *x*‐axis label UO.

**Figure 3:**
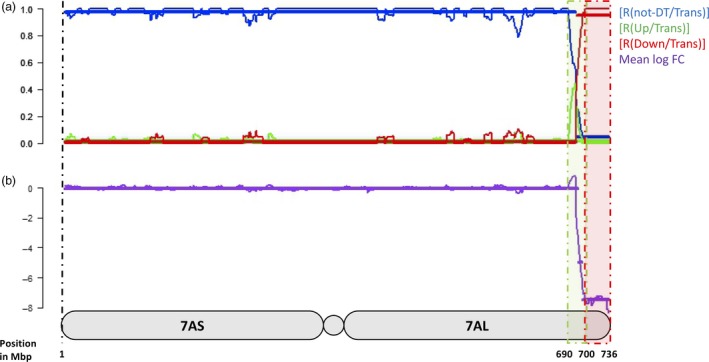
The chromosomal spread of 7A genes showing altered transcription in CS + 7HL. (a) The ratio of not‐DT to transcribed genes [R(not‐DT/Trans)], the ratio of up‐regulated to transcribed genes [R(Up/Trans), the ratio of down‐regulated to transcribed genes [R(Down/Trans)]. (b) The mean log FC in CS + 7HL and CS along the 7A chromosome. Each segment is represented by a horizontal bar along each measure (change point positions given in Mbp as positions on the 7A pseudomolecule.

Four measures were used to quantify the distribution of DT in the wheat genome and along the 7HL chromosome arm; these were the ratio between the number of nondifferentially transcribed (‘not‐DT’) and the total number of transcribed genes [R(not‐DT/Trans)], the ratios between the number of up‐ or down‐regulated and the total number of transcribed genes [R(Up/Trans) and R(Down/Trans)] and the mean log fold change (mean log FC). For the 7HL arm, the R(not‐DT/Trans) measure indicated that the central segment of the arm was enriched for not‐DT genes (Figure 1). The R(Up/Trans) measure was highest at the centromere (0.38) and lowest at the telomere (0.12), while the R(Down/Trans) measure behaved in the opposite fashion (0.15 at the centromere and 0.33 at the telomere). As a result, the mean log FC parameter was at its most positive at the centromere (+0.56) and at its most negative at the telomere (−0.78). Overall, the proximal region of the arm was enriched for up‐regulated genes and the distal region for down‐regulated ones, leaving the interstitial region neutral. A similar analysis was conducted for the wheat gene complement. On average, a similar number of genes per chromosome were down‐regulated (24) as were up‐regulated (20), but the numbers were particularly high on chromosome 7A (117 down‐ and 29 up‐regulated genes) (Figure 2). The distribution of DT genes along chromosome 7A is shown in Figure 3. A major feature was the behaviour of all four parameters in two contiguous regions in the distal part of the chromosome's long arm. The first region, a ~10 Mb stretch from 690 to 699 Mbp, harboured 27 transcribed genes, of which 12 were DT (all up‐regulated). Along the segment, R(Up/Trans) increased from 0.01 to 0.33, and the mean log FC was +0.52, reflecting a higher average transcript abundance [mean fragments per kilobase of transcript per million mapped read (FPKM): 14.59]. The second region represented the most distal 36 Mbp of the arm (700–736 Mbp), in which there was a set of down‐regulated genes, resulting in a R(Down/Trans) value of 0.98, a R(not‐DT/Trans) value of just 0.03 and a R(Up/Trans) value of zero. Of the 101 genes present in this region and transcribed in CS, 99 were down‐regulated in CS + 7HL and the other two were not‐DT genes. The associated mean log FC value was −8.21, and the average transcript abundance in CS + 7HL was extremely low (mean FPKM: 0.25); 25 of the genes recorded an FPKM of zero in every replicate.

### The terminal region of 7AL in CS + 7HL harbours a 36 Mbp deletion

**Figure 4:**
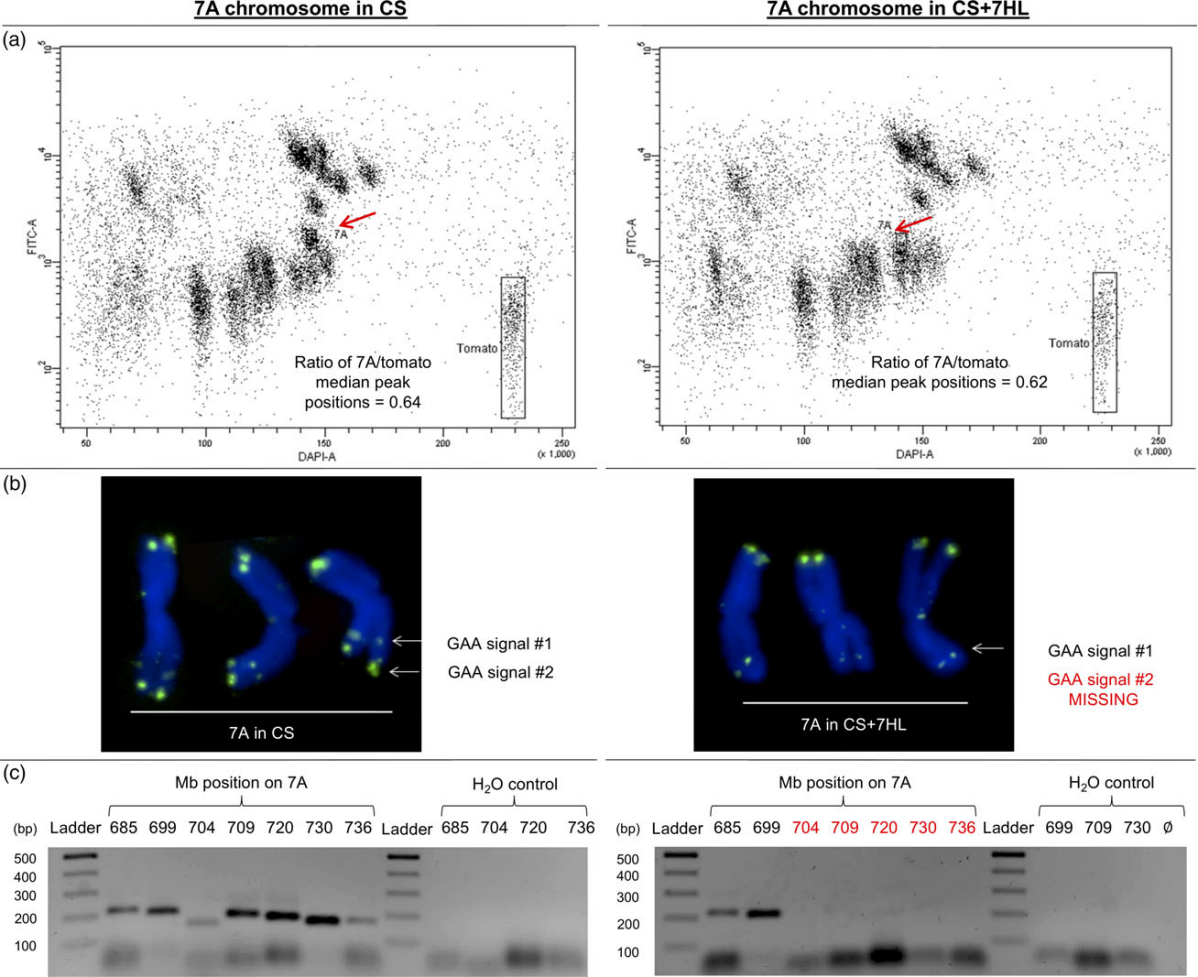
The 7AL arm in the CS/B addition lines carries a major deletion. The deletion is apparent given (a) a size difference in the 7A copies flow‐sorted from CS and CS + 7HL, (b) the loss of a GAA FISH site (green) at the distal end of the chromosome and (c) the PCR‐based genotyping of 7A deleted region on genomic DNA from CS and CS + 7HL.

A comparison of the physical length of flow karyotyped mitotic copies of chromosome 7A suggested that the version harboured by CS + 7HL was approximately 3.0% shorter than the one harboured by CS (Figure 4a). When GAA satellite‐based FISHIS patterns of the two versions of chromosome 7A were compared, it was apparent that the most terminal GAA signal on CS 7AL was not present on CS + 7HL 7AL (Figure 4b). The estimated centromeric index (ratios of the long arm to the short arm) of the two versions of 7A revealed that while CS copy is metacentric, CS + 7HL copy was submetacentric. To support the strong indication that the two copies differed as a result of a large deletion event, a set of chromosome 7A molecular markers was developed spanning the 685–736 Mbp region of 7AL (Table S2). When these were used to genotype CS and CS + 7HL, the results confirmed the loss in the latter line of a region between 700 and 736 Mbp of 7A (Figure 4c). The same deletion was also found to be present in each of the six CS/B whole chromosome addition lines, as confirmed by C‐banding (Figure S1).

### The distal deletion on 7AL does not induce DT of homeologous genes

**Figure 5:**
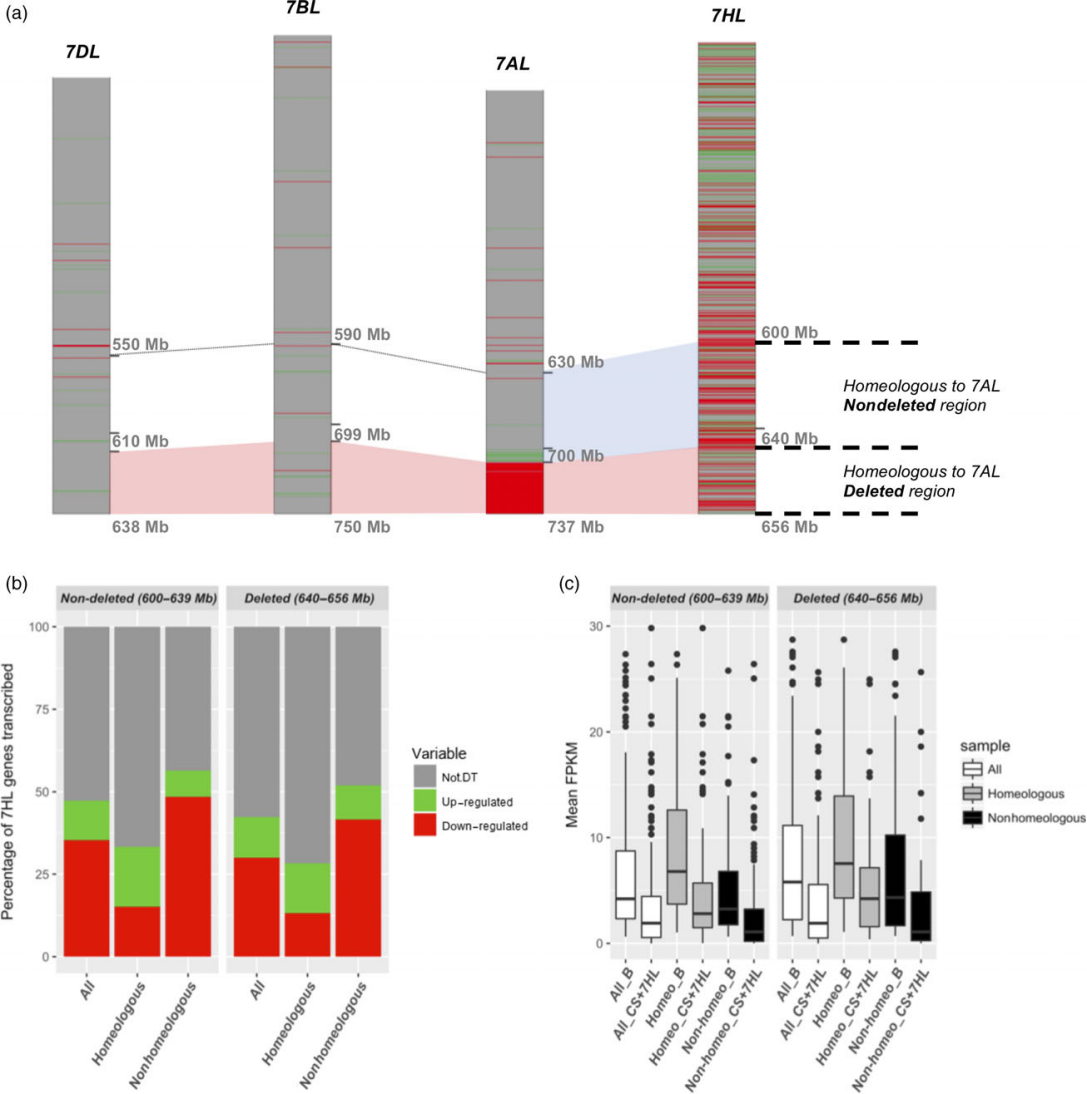
Compensation for genes deleted in the 7A copy present in CS + 7HL by their homeologs in 7B, 7D and 7HL. (a) (Non)‐compensation at the level of transcription, (b) the distribution of not‐DT, up‐ and down‐regulated genes in the whole (All), homeologous and nonhomeologous portions of 7HL chromosome arm homeologous to the nondeleted (7HL positions 600–639 Mbp) or deleted 7AL region (7HL positions 640–656 Mbp), (c) Boxplot representation of transcript abundance (FPKM) of All, homeologous and nonhomeologous 7HL genes present in CS + 7HL and in B mapping to the regions homeologous to either the nondeleted or deleted regions of 7AL.

The regions on chromosome arms 7BL (699–750 Mbp) and 7DL (610–636 Mbp) are homeologous to the 7AL deletion harbour, respectively, 143 and 122 genes. Of these, just six of the 7B genes and two of the 7D ones were transcribed at a higher level in CS + 7HL than in CS (Figure 5a). The R(DT/Trans) values are consistent with the value of ca. 3% which pertains elsewhere in the genome. There was, therefore, no apparent effect of the deletion on the transcription of the 7B and 7D homeologs. The homeologous segment of 7HL (640–656 Mbp) harbours 130 transcribed genes, of which 55 were classified as DT genes in the contrast B *vs* CS + 7HL. As described above (Figure 1), the proportions of DT and not‐DT genes in this region did not differ significantly from those obtained in its proximally adjacent segment (600–639 Mbp), which harbours a similar gene density (167 genes, of which 79 were classified as DT genes). Using stringent sequence comparison parameters and collinearity guided strategy, we defined a set of 7HL genes homeologous to 7AL and selected 1 : 1 relationships to investigate whether the loss of 7AL genes induced DT of 7HL syntenic genes in CS + 7HL. The transcription of the homeologous (40%) and nonhomeologous (60%) genes in both the deleted and the nondeleted regions showed similar distributions of homeologs/nonhomeologs of not‐DT (50/50, ±0.47), up‐regulated (55/45 ± 7.07) or down‐regulated (17/83, ±0.7) genes (Figure 5b). The abundance of transcript from the homeologous genes was greater than that from the nonconserved ones, which was the case for both the deleted and the nondeleted regions: the median FPKM for homeologous genes in the deleted region was 10.53 in B and 4.89 in CS + 7HL; for the nonhomeologous genes, the median FPKMs were, respectively, 5.80 and 1.11 (Figure 5c). Overall, therefore, there was no effect of the deletion on the level of transcription of the 7HL gene complement, although a large proportion of the 7HL segment harboured homeologs of the genes lost as a result of the deletion.

### The distal deletion on 7AL is partially compensated for by orthologs on 7HL

**Figure 6:**
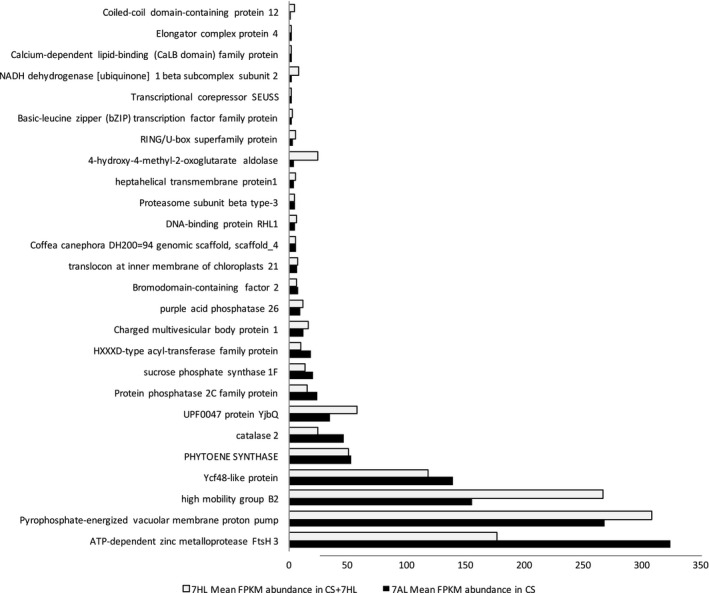
Partial compensation at the level of transcription for genes deleted in the 7AL copy present in CS + 7HL by their orthologs in 7HL. The partial compensation (at least 50%) of 26 genes deleted in the 7AL chromosome present in CS + 7HL is represented by the abundance given as mean FPKM across all bio‐replicated of the 7AL gene copy in CS (dark blue) and the 7HL ortholog in CS + 7HL (clear blue). On *y*‐axis is given the functional annotation of the barley 7HL genes according Mascher *et al*. (2017).

Of the 923 7HL genes transcribed in B, 702 (76%) were transcribed at a detectable level in CS + 7HL: 375 transcripts were represented by a count of 1–5 FPKM and 327 by >5 FPKM; of the latter, 92 were strongly transcribed (20–1360 FPKM). Around 60% of these genes had at least one transcriptionally active wheat homeolog, so are likely to contribute to the transcript pool. Among the set of highly transcribed 7HL genes, 47 were homeologs of a 7AL gene deleted in CS + 7HL; of these, 26 were at least half as abundantly transcribed in CS + 7HL as was their 7AL homeolog in CS (Figure 6). The indication was, therefore, that the presence of 7HL partially compensated for the loss of transcripts due to the deletion event on 7AL.

### Biological relevance of alterations in the pattern of wheat and barley gene transcription

A gene ontology (GO) analysis of the set of wheat DT genes showed that among the 451 up‐regulated genes, there was an enrichment for 174 terms for biological processes and molecular functions, relating mostly to the response to biotic, abiotic and oxidative stresses. In particular, the GO terms ‘immune system process’, ‘response to biotic stimulus’, ‘immune response’ and ‘ubiquitin‐like protein ligase binding’ were prominent, as were terms relating to secondary metabolism synthesis such as ‘phenylpropanoid metabolic process’ and ‘secondary metabolite biosynthetic process’ (Table S3). Meanwhile, the 410 down‐regulated genes strongly featured those relating to energy production, such as ‘cellular response to starvation’, ‘glycolipid biosynthetic process’ and ‘liposaccharide metabolic process’ (Table S4). The implication was that the alterations to the wheat transcriptome induced by the presence of the barley telosome are nonrandom, perhaps reflecting a response to a loss in homoeostasis at the protein and metabolite level. A similar analysis was also applied to the set of genes present in the 7AL deletion, but no significant GO term enrichment was revealed. When the up‐regulated set of 156 7HL genes was analysed, there was a significant enrichment for 26 GO terms, notably ‘protein synthesis’, ‘peptide metabolic process’, ‘translation and amide biosynthetic process’ (Table S5). The set of 233 down‐regulated 7HL genes were enriched for 14 GO terms, potentially associated in some way with sexual incompatibility: the predominant terms were ‘cell recognition’, ‘single organism reproductive process’, ‘reproduction’, ‘multiorganism process’ and ‘multicellular organismal process’ (Table S6). Overall, the GO term enrichment analysis of the DT genes indicated that the altered transcription of recipient and donor genes in CS + 7HL affected a subset of biological functions.

## Discussion

The RNA‐seq platform was used here to capture the transcriptomes of CS, CS + 7HL and B, and applied a more stringent set of criteria that is normally used for mapping reads to a genomic sequence. This was thought necessary to ensure discrimination between homeologs. Rather than aiming to maximize sequencing depth, it was considered a better use of resources to increase the level of biological replication. The approach allowed the transcript abundance of over 35 000 wheat and nearly 1000 barley (7HL) genes to be estimated in the CS + 7HL line, representing, respectively, 31.8% and 34.5% of the annotated gene content. The replication also provided a high measure of confidence to the identification of the 960 wheat and 389 barley genes classified as DT genes in two contrasts CS *vs* CS + 7HL and B *vs* CS + 7HL.

### The introgression of 7HL into wheat altered the recipient's and donor's transcriptomes to a varying extent

Wide hybridization is often associated with profound rearrangements of the host and exotic species chromosomal structure, along with alterations to both the DNA sequence and the epigenome; ultimately, these have a profound effect on the transcriptome of the wide hybrid (Buggs *et al*., 2011; Chen and Ni, 2006; Comai, 2000; Comai *et al*., 2003; Feldman and Levy, 2005, 2012; Feldman *et al*., 1997; Gernand *et al*., 2005; Kashkush *et al*., 2002; Levy and Feldman, 2004; Pumphrey *et al*., 2009; Xu *et al*., 2014). With respect to the CS/B cross, it was not possible to stabilize the initial allohaploid by chromosome doubling, as has been achieved for a range of other wheat‐based wide crosses, most notably in the wheat/rye combination triticale. At the time the CS‐B hybrid was made (Islam *et al*., 1981), large‐scale transcriptomic analyses were not feasible, and as a result, the impact of genomic shock could not be investigated at this level, although it is known that the early back‐cross generations following the production of the CS/B allohaploid were highly abnormal phenotypically (Islam *et al*., 1981). Based on the experience with stabilizable wide hybrids, it is more than probable that combining two such distantly related species as wheat and barley would have induced major disruptions to the transcriptome, affecting both the barley and wheat components. The present study has revealed, however, that only 3% of wheat genes showed any evidence of altered transcription in the CS + 7HL line, while ~42% of the barley genes on 7HL were affected in this way. The relatively mild effect of the presence of 7HL on the wheat transcriptome probably reflects the stabilizing effect of the two generations of back‐crossing imposed to reduce the barley complement present in the founding CS/B allohaploid to a single chromosome and to restore that of wheat to its euploid state (Islam, 1983; Islam *et al*., 1981). In contrast, a large proportion of the genes on 7HL appear to have experienced DT, much of which was likely induced by the genomic shock occurring within the founder allohaploid and its immediate progeny. Although the barley genes affected are distributed along the entire length of the 7HL arm, the ratio of genes experiencing DT proved to be higher at the arm ends: at the proximal end, a majority of the genes affected were up‐regulated, while at the distal end, the majority was down‐regulated. A small number (13) of the barley genes have been fully silenced, suggesting a lack of the necessary regulatory sequences; for the rest, their altered transcription intensity implies either the absence of a fully functional regulatory environment and/or an effect of gene dosage due to the presence of their wheat homeologs. Many (~76%) of the barley genes were highly transcribed in the CS + 7HL line, although the comparison with their transcription in B reveals various degrees of regulation in the wheat recipient background. The evidence suggests that most of the genes present on 7HL are regulated by wheat sequences, although for some, this heterologous regulation appears unable to exert the same level of control as managed by native sequences within a barley context. There is no evidence supporting dominance over their transcription exerted by any of the three wheat subgenomes.

### The biological relevance and regulation DT genes

The genes which were differentially transcribed were distributed throughout the wheat genome and along the entire length of chromosome 7HL, implying a level of stochasticism. However, when subjected to a GO analysis, there was evidence for nonrandomness, as only a subset of biological functions was affected. Many of the up‐regulated genes on barley 7HL were associated with protein translation, synthesis and modification, while on the other hand, the down‐regulated ones were related to reproduction and sexual compatibility, encoding a range of transcription factors, hormone receptors, receptor‐like protein kinases and certain stress‐related proteins. Notably, many of the barley 7HL genes which were up‐regulated in the CS + 7HL line encoded products controlling cellular basal functions such as protein synthesis, whereas the down‐regulated genes encoded products involved in reproduction and adaptation; the latter genes may contribute to the reduced fertility of the CS + 7HL line in comparison with the other CS‐B ditelosomic addition lines described by Islam (1983). The data indicate that regulatory elements within the wheat genome exert at best partial control over the transcription of the 7HL genes, possibly reflecting the length of the time which has passed since the evolutionary divergence of barley from wheat. The polarized regulation of barley gene transcription (with the transcription of genes at the 7HL telosome's proximal end tending to be favoured) observed in CS + 7HL could arise from the status of telosome's chromatin being unusual or may be a consequence of topology of the telosome within the interphase nucleus (Branco and Pombo, 2007; Elcock and Bridger, 2010; Schubert *et al*., 2014). The present data set is inadequate to address this issue, as it is limited to a single tissue and developmental stage, while the topology of 7HL in the addition line nucleus is hard to ascertain (Grob *et al*., 2014; Mukhopadhyay *et al*., 2013; Schubert *et al*., 2014). Further transcriptomic, epigenetic and spatial analyses of the host and alien chromosomes in other tissues and developmental stages would be needed to reveal the mechanisms impacting the donor and recipient gene transcription in the CS + 7HL line.

### The CS + 7HL addition line harbours a large deletion in 7AL

The CS/B addition lines have been used to identify the genetic basis of a number of traits (Ashida *et al*., 2007; Bilgic *et al*., 2007; Kato *et al*., 2008; McCallum *et al*., 2004; Sakai *et al*., 2009; Shi and Endo, 1997; Tang *et al*., 2011; Yuan *et al*., 2002). Here, it has been demonstrated that they all share a large (36 Mbp) deletion at the distal end of 7AL. According to the annotation of the 700–736 Mbp region of the 7A pseudomolecule (IWGSC, 2018), the deletion has resulted in a loss of >500 genes. A similarly sized deletion in this region has also been identified among the products induced by the action of a gametocidal chromosome (Endo and Gill, 1996). The fact that the deletion is also present in all six of the CS/B disomic addition lines implies that it could not have arisen at the same time as the formation of the 7HL telosome. Rather, it must have already been present during the development of the addition lines. Islam *et al*. (1981) state that, although 19 independent CS/B allohaploids were produced, only one (which harboured the expected chromosome number of 28) was used for back‐crossing to CS to produce the disomic (and later the ditelosomic) addition lines. The most plausible scenario is therefore that the deletion was fixed in the CS stock used as the recurrent back‐cross parent to develop the addition lines. At the level of karyotype, the size difference between the wild‐type and the deletion‐harbouring copy of chromosome 7A is not easy to identify. At the time when the addition lines were created, there was neither any understanding of the ubiquity of deletions in wheat nor had marker technology developed to a sufficient level to allow for such a deletion to have been identified via genotyping. In the meantime, it has been recognized that CS is not a homogeneous genotype (Mott and Wang, 2012). As demonstrated by Kubaláková *et al*. (2002), the bread wheat genome is known to harbour a variety of translocations, deletions and duplications, some of which arise as a result of the genomic shock caused by interspecific hybridization (Bento *et al*., 2008; Feldman *et al*., 1997; Fu *et al*., 2013; Liu *et al*., 1998). Thus, the performance of individual addition and other introgression lines may be affected not just by the presence of the donor chromosome(s) or chromosome segment(s), but also by hidden variation in the karyotype of the recipient species.

## Conclusions

This comparative analysis of transcription in bread wheat, barley and the wheat–barley 7HL addition line has identified that the addition line transcriptome had some unexpected features. The cluster of down‐regulated wheat genes on chromosome 7A was shown to be due not to an interaction with the barley telosome, but instead was revealed to be due to a 36 Mbp deletion lying at the distal end of the long arm, likely present in the stock of CS used to generate the disomic addition line set. The genes experiencing DT in the CS + 7HL line were randomly distributed in terms of their genomic location, but appeared to be nonstochastic in relation to their function. This set of genes provides a resource for investigating the molecular basis of DT, which may involve regulatory sequences, epigenetic changes or the organization of chromosome domains in the interphase nucleus. These analyses will be facilitated by the availability of high‐quality wheat and barley genome sequence and its associated annotation. The present study has provided a set of genes of potential relevance for determining the gene networks responsible for interspecific incompatibility between wheat and its related species.

## Materials and methods

### Sample preparation and sequencing

The plant material used in this study comprised CS wheat (2n = 6x = 42, AABBDD), Betzes barley (B; 2n = 2x = 14, HH) and the CS/B addition line harbouring a pair of 7HL telosomes (CS + 7HL; AABBDD + 7HL′′). Grain of CS + 7HL and of the six viable whole chromosome CS/B addition lines (+2H through +7H) were obtained from the National BioResource Project, Komugi (shigen.nig.ac.jp/wheat/komugi/strains/nbrpDetailAction.do?strainId=LPGKU2105). The mitotic chromosome number of each aneuploid line was checked to confirm the presence of the expected chromosome complement, following the Kopecký *et al*. (2007) procedure. Plants were raised under a 16 h photoperiod and a day/night temperature regime of 20 °C/16 °C. When they had reached Zadoks stage 11 (first leaf emerged), the oldest leaf from each of three plants of each line was pooled into a single sample (~25 mg), snap‐frozen in liquid nitrogen and held at −80 °C. Six biological replicates were collected for each line. RNA was extracted using an RNeasy Plant Mini Kit (Qiagen, Hilden, Germany) and treated with RNase‐free DNase I (Qiagen) to remove contaminating genomic DNA. The integrity of the RNA was assessed using a 2100 Bioanalyzer (Agilent Technologies, Santa Clara, CA), and an RNA integrity number (RIN) was calculated. Only samples with a RIN >7 were retained for library construction and sequencing, carried out at POPS‐IPS2 platform (http://www.ips2.u-psud.fr/spip.php?article213). cDNA libraries were generated for each sample using a TruSeq RNA Sample Preparation Kit (Illumina, San Diego, CA). The samples were randomly pooled by six and sequenced on 2 × 100 bp paired‐end flow cells using HiSeq2000 device (Illumina), generating ~30 million reads per sample. Ribosomal RNA sequences were removed with the help of sortMeRNA program (Kopylova *et al*., 2012), reads were cut after the first uncalled base (‘N’) and low‐quality reads (Phred score <Q30) were discarded using Trimmomatic software (Bolger *et al*., 2014).

RNA‐seq data have been deposited in the sequence read archive under the BioProject accession number PRJNA412872.

### Transcript analysis

Each of the reads for each of the 18 samples (six replicates of each of CS, B and CS + 7HL) was mapped against the ‘High Confidence’ coding sequences of both wheat (IWGSC RefSeq v1.0, see IWGSC, 2018) and barley (IBSC RefSeq, see Mascher *et al*., 2017) using Bowtie v1.0.0 software (Langmead *et al*., 2009) with no mismatches allowed. The quantification of transcripts abundance was performed using RSEM v1.2.8 software (Li and Dewey, 2011). The assignment of DT was based on two comparisons: one between CS and CS + 7HL, and the other between B and CS + 7HL. Transcripts recording a count of less than one per million mapped reads were ignored, and the counts were then normalized by the effective library size using the trimmed mean of the logarithm of the transcript abundance ratios (TMM); DT was identified using edgeR package version 3.18.1 (Robinson *et al*., 2010) of the R software version 3.4.1, based on a generalized linear model where the log of the mean expression is a linear function of the factor ‘group’ defining all biological replicates of either CS, B or CS + 7HL. The false discovery rate for calling DT was set as 5%. Up‐/down‐regulation refers to genes for which the transcript abundance was significantly higher/lower in CS + 7HL than in either CS or B, without applying fold change threshold to see all changes. Finally, the TMM‐normalized counts were converted into FPKMs to enable comparisons between different genes within or between samples.

### Genomic distribution of the genes responsible for the transcripts

The density of the transcribed genes was calculated on the basis of a sliding 10 Mbp window, with a step of 1 Mbp along each chromosome. Similarly, the ratios of down‐ and up‐regulated to transcribed transcripts [R(Down/Trans), R(Up/Trans)] were calculated. The mean logarithm of the fold change in transcript abundance (logFC) in the comparisons CS *vs* CS + 7HL and B *vs* CS + 7HL was determined for the same genomic windows. Each of these measures was processed using the R package changepoint v2.2.2 program (Killick and Eckley, 2014) employing the ‘Pruned Exact Linear Time’ method (Killick *et al*., 2012).

### Flow cytometry

The procedure used for preparing metaphase chromosome suspensions and subsequent FISHIS labelling has been described by Vrána *et al*. (2016). A crude suspension of tomato nuclei obtained from chopped leaf was added to the chromosome suspensions to serve as an internal reference for the estimation of the DNA content of chromosome 7A in both CS and CS + 7HL. Flow cytometry and sorting were conducted using a FACSAria flow cytometer (BD Biosciences, San José, CA) equipped with blue laser (488 nm, 100 mW) for FITC fluorescence excitation and a UV laser (355 nm, 100 mW) for DAPI fluorescence excitation.

### Cytogenetic analysis

Chromosomes 7A sorted from CS and CS + 7HL were subjected to FISH, using as probes either a GAA microsatellite or an Afa family repeat, to identify individual chromosomes. The FISH protocol followed that of Vrána *et al*. (2016), except that in case of metaphase spreads, the denaturation period was extended to 2 min at 80 °C. C‐banding analysis was performed following the protocol previously described by (Endo, 2011).

### Genotypic analysis

Genes present on the group 7 chromosomes for which there were known to be a full set of three homeologs were identified using the homeologs data set provided with the IWGSC RefSeq_v1.0 (IWGSC, 2018). The genomic sequence of each homeolog was processed using GSP software (Wang *et al*., 2016) to allow PCR primers to be designed with a Tm of 50–65 °C and a GC content of 50%–60%; the predicted amplicon size range was limited to the range 150–250 bp. The selected primer sequences were scanned against both the IWGSC RefSeq (IWGSC, 2018) and IBSC RefSeq (Mascher *et al*., 2017) sequences to remove any which were likely nonspecific for the 7A homeolog. Finally, PCRs were run on a set of templates (genomic DNA from CS and CS + 7HL.

### Definition of homeologous and nonhomeologous gene sets

Sets of homeologous and nonhomeologous genes between 7HL and wheat subgenomes were determined by whole genome comparison of protein sequences using BLASTp (Altschul *et al*., 1990) leveraged by MCScanX software (Wang *et al*., 2012) with *E*‐value threshold of 10e‐5 and a size of synteny block of 10 genes. Finally, only 1 : 1 relationships were retained for each subgenome comparison.

### Gene ontology term enrichment analysis

Gene ontology terms were associated with each of the wheat and barley genes according to functional annotations provided by, respectively, IWGSC (2018) and Mascher *et al*. (2017). Ontologizer2.0 software (Bauer *et al*., 2008) was applied to identify GO term enrichment. The full set of transcribed genes for wheat and 7HL genes was used as reference comparison set for the enrichment analysis of the genes deleted in 7AL and for the up‐ and down‐regulated genes in CS + 7HL. *P*‐values were evaluated using the Parent–Child–Union method (Grossmann *et al*., 2007) and then adjusted by applying the Benjamini–Hochberg correction (Benjamini and Hochberg, 1995). GO terms considered as enriched were retained on the basis of adjusted *P*‐value thresholds of 0.01 (wheat) and 0.05 (7HL).

## Conflict of interest

The authors declare no conflict of interest.

## Supporting information


**Figure S1** All CS/B addition lines carry a major deletion in the 7AL chromosome arm. The same deletion as detected in the CS + 7HL ditelosomic addition line is present in the six CS/B whole chromosome addition lines, as shown by C‐banding. Chromosomes 7A are indicated on the pictures by red arrows.

**Table S1** The RNA‐seq mapping fitness.
**Table S2** Molecular markers used to genotype 7AL deletion in CS and CS + 7HL.
**Table S3** The gene ontology terms enriched in the set of wheat up‐regulated genes in CS + 7HL.
**Table S4** The gene ontology terms enriched in the set of wheat down‐regulated genes in CS + 7HL.
**Table S5** The gene ontology terms enriched in the set of barley 7HL genes up‐regulated genes in CS + 7HL.
**Table S6** The gene ontology terms enriched in the set of barley down‐regulated genes in CS + 7HL.
