# Supplementary figures and images for: Transcriptome reprogramming due to the introduction of a barley telosome into bread wheat affects more barley genes than wheat
Source: Plant Biotechnol J. 2018 Apr 13;16(10):1767–77. doi: 10.1111/pbi.12913 (PMC6131412; doi:10.1111/pbi.12913)

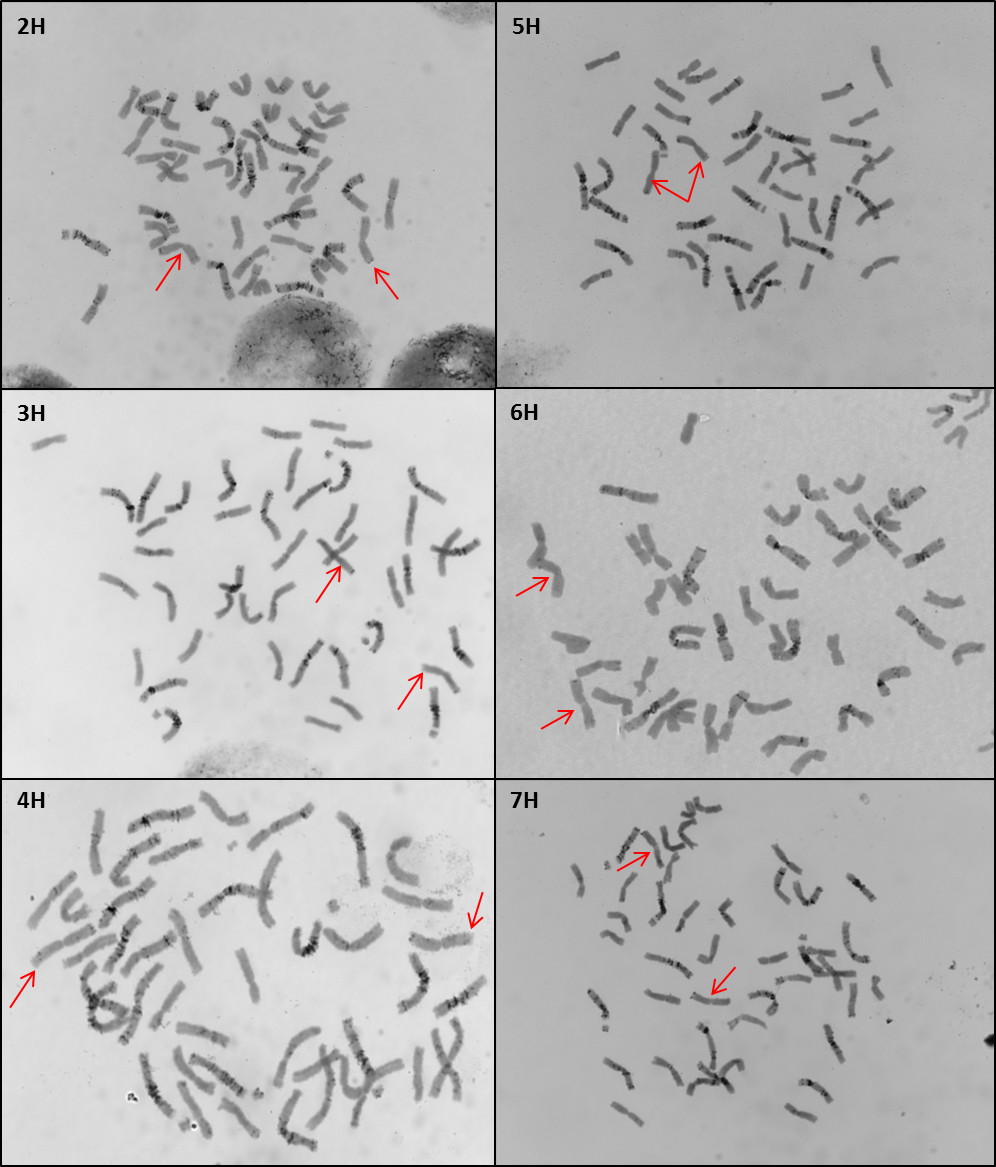


Fig S1: All CS/B addition lines carry a major deletion in the 7AL chromosome arm.

Supplement: Supplementary file 1 — Figure S1 All CS/B addition lines carry a major deletion in the 7AL chromosome arm. The same deletion as detected in the CS + 7HL ditelosomic addition line is present in the six CS/B whole chromosome addition lines, as shown by C‐banding. Chromosomes 7A are indicated on the pictures by red arrows. [file PBI-16-1767-s001.docx]
